# *Helicobacter cinaedi* bacteremia with cellulitis in a living-donor kidney transplant recipient identified by matrix-assisted laser desorption ionization time-of-flight mass spectrometry: a case report

**DOI:** 10.1186/s13104-017-2403-5

**Published:** 2017-02-07

**Authors:** Ai Katsuma, Izumi Yamamoto, Yukiko Tsuchiya, Mayuko Kawabe, Takafumi Yamakawa, Haruki Katsumata, Aki Mafune, Yasuyuki Nakada, Akimitsu Kobayashi, Kentaro Koike, Akihiro Shimizu, Yudo Tanno, Ichiro Ohkido, Nobuo Tsuboi, Seiji Hori, Hiroyasu Yamamoto, Takashi Yokoo

**Affiliations:** 1 Division of Nephrology and Hypertension, Department of Internal Medicine, The Jikei University School of Medicine, 3-25-8, Nishi-Shimbashi, Minato-ku, Tokyo, 105-8461 Japan; 2 Department of Infectious Diseases and Infection Control, The Jikei University School of Medicine, Tokyo, Japan

**Keywords:** *Helicobacter cinaedi*, Cellulitis, gyr-B PCR, MALDI-TOF MS, Case report

## Abstract

**Background:**

*Helicobacter cinaedi* causes bacteremia and cellulitis, mainly in immunocompromised patients. We report a rare case of *H. cinaedi* bacteremia with cellulitis in a living-donor kidney transplant recipient identified by matrix-assisted laser desorption ionization time-of-flight mass spectrometry (MALDI-TOF MS).

**Case summary:**

A 54-year-old Asian man with IgA nephropathy underwent living-donor kidney transplantation 14 years previously. He was admitted to our hospital for evaluation of fever and multifocal cellulitis. *H. cinaedi* was isolated and identified from the patient’s blood using matrix-assisted laser desorption ionization time-of-flight mass spectrometry and gyrase subunit B-targeted polymerase chain reaction assays. Matrix-assisted laser desorption ionization time-of-flight mass spectrometry has proven over the years to be a rapid and accurate universal method for the identification of microorganisms.

**Conclusions:**

The combined use of these detection methods enabled the appropriate administration of 6 weeks of antibiotic therapy. The patient recovered completely, with no recurrence.

## Background

*Helicobacter cinaedi* is a gram-negative enterohepatic spiral bacillus found in the digestive tracts of humans and animals (e.g., dogs, cats, and hamsters). This organism was first isolated from rectal cultures from homosexual men in 1984 [1]. Since that time, *H. cinaedi* infections have been detected from blood and stool samples obtained mainly from immunocompromised patients [2]. The first case of *H. cinaedi* infection in Japan in an immunosuppressed patient was reported in 2003 [3]. However, recent reports have demonstrated that this organism can cause nosocomial infections in immunocompetent patients after orthopedic surgery [4]. The reported detection rate of *H. cinaedi* among positive blood cultures (i.e., blood samples with any positive culture result) in Tokyo, Japan is 0.22% [5]. The clinical manifestations of *H. cinaedi* infection include fever, enterocolitis, diarrhea, cellulitis, arthritis, meningitis, and recurrent bacteremia. Isolation of *H. cinaedi* requires a long incubation period in automated blood culture, and this pathogen is sometimes resistant to macrolides and quinolones [6]. For these reasons, *H. cinaedi* infections tend to be overlooked, are likely to recur, and often require long-term antibiotic therapy. In addition, data on *H. cinaedi* infection in kidney transplant recipients are very limited; only five cases have been reported to date [3, 7]. Several genomic methods, such as 16S rRNA sequencing and polymerase chain reaction (PCR), can be used to identify *H. cinaedi*. Matrix-assisted laser desorption ionization time-of-flight mass spectrometry (MALDI-TOF MS) is a new technology for routine identification of bacteria in clinical microbiology laboratories. Here, we report a case of *H. cinaedi* bacteremia with cellulitis in a living-donor kidney transplant recipient identified by MALDI-TOF MS, with a review of the related literature.

## Case presentation

**Fig. 1:**
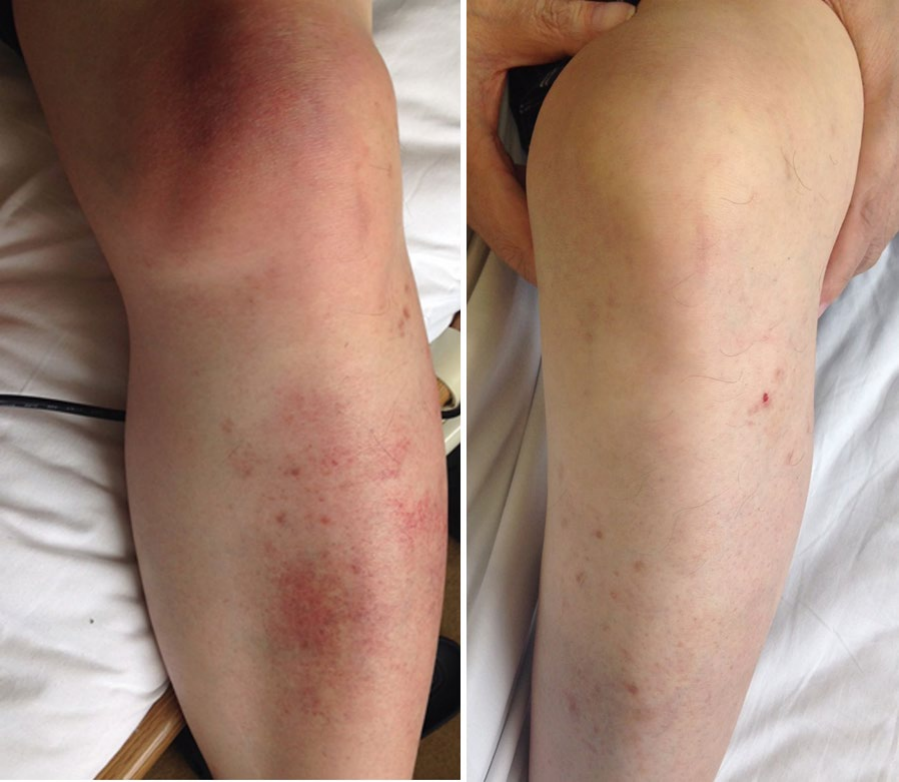
*Left*, cellulitis in the left lower leg and knee. Local swelling with salmon-pink skin discoloration and local heat with spontaneous pain was evident. *Right*, left leg after 6 weeks of antibiotic therapy

**Fig. 2:**
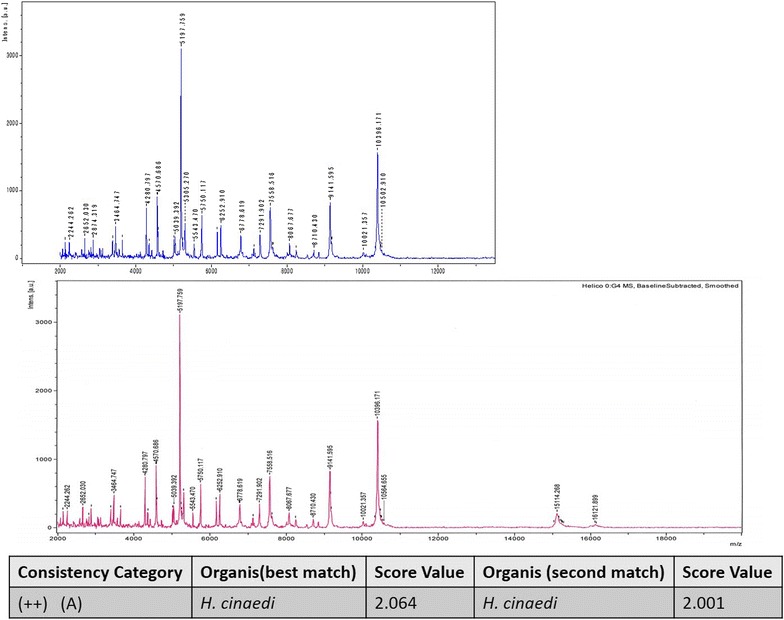
Intact-cell mass spectrometry profiling using matrix-assisted laser desorption ionization time-of-flight mass spectrometry. *Upper panel*, intact-cell mass spectrometry profiles from our case; *lower panel*, the reference case. Table at *bottom* shows the identification score using the integrated pattern-matching algorithm of the MALDI Biotyper 3.0 software^®^ (Bruker Corp., Germany). The patient’s identification score was 2.064. A score of ≥2.0 is useful for identification to the species level

**Fig. 3:**
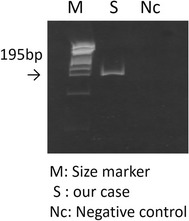
Detection of *Helicobacter cinaedi* in blood using gyrase subunit B-targeted polymerase chain reaction. *M* molecular size marker, *Nc* negative control: distilled water (DNA- and DNase-free water). Polymerase chain reaction was performed using the forward primer AGGGATTCCACAAAGTGAGC and the reverse primer TCTTGTCCTGTGCGTTCATC. This resulted in a product of 195 base pair

**Fig. 4:**
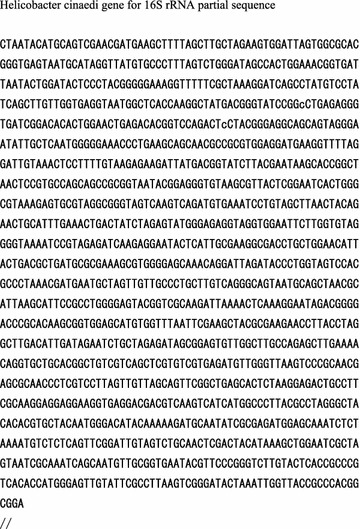
Partial sequence of the *Helicobacter cinaedi* 16S rRNA gene

A 54-year-old Asian man with IgA nephropathy underwent living-donor kidney transplantation 14 years previously. His medical condition had been almost stable for 14 years. He developed multifocal salmon-pink skin discoloration, and swelling and spontaneous pain in the left knee and leg. The symptoms had gradually expanded across the right forearm and outside of the right thigh. He was admitted to our hospital for the evaluation of fever (39 °C) and multifocal cellulitis (Fig. 1). Two months before admission, he had developed chronic diarrhea. His serum creatinine level was stabilized at 1.7 mg/dL with maintenance immunosuppressive therapy comprising tacrolimus (3 mg/day), mycophenolate mofetil (1500 mg/day), and prednisone (4 mg every other day). The tacrolimus trough concentration was 6.3 ng/mL. The patient had been a dog breeder for 12 years. On admission, his white blood cell count was 12,400/μL and his C-reactive protein level was 3.9 mg/dL. The patient was initially treated with ampicillin/sulbactam (9 g/day intravenously). Two days after the initiation of this therapy, he was afebrile. Four days later, an automated blood culture (Bactec FX^®^; Becton–Dickinson and Company, Sparks, MD, USA) showed positivity for gram-negative spiral bacilli. A colony obtained from the patient’s blood culture was analyzed by MALDI-TOF MS (Biotyper ver. 3.0^®^; Bruker Corporation, Germany). The identification score was 2.064 (>2.0), indicating accurate identification of *H. cinaedi* (Fig. 2). Additional evaluation of the patient’s blood specifically for *H. cinaedi* by means of gyrase subunit B (gyrB)-targeted PCR assays (using the forward primer AGGGATTCCACAAAGTGAGC and the reverse primer TCTTGTCCTGTGCGTTCATC to amplify the region of the *gyrB* gene) yielded positive results. We performed gyrB-targeted PCR using a single colony isolated from a blood culture. We used distilled water (DNA- and DNase-free water) as a negative control to prevent contamination of gyrB-targeted PCR (Fig. 3). In addition, we performed 16S rRNA gene sequencing of this strain, and the results were > 99% consistent with the existing sequence (Fig. 4). Given these results, we diagnosed the patient with *H. cinaedi* bacteremia with cellulitis. We examined the genomic heat shock protein (HSP) 60 sequence of the blood culture isolate, which resulted in identification of cluster B *H. cinaedi*. A blood culture obtained at 11 days was positive for *H. cinaedi*, but a culture obtained at 15 days was negative. The patient’s cellulitis gradually resolved. The patient continued antibiotic treatment for a total of 6 weeks (ampicillin-sulbactam in a drip for 2 weeks and oral levofloxacin for 4 weeks), and he had no recurrence 6 months after this therapy. His stool culture was negative, although it was taken after treatment. We did not apply enteric bacteria elimination in this case.

## Discussion

*Helicobacter cinaedi* infection in kidney transplant recipients remains poorly understood. Only six cases, including our case, have been reported to date. All six cases had a positive blood culture, and four of these patients had cellulitis [3, 7]. The correlation between *H. cinaedi* infection and kidney transplantation may be due to the use of immunosuppressive agents. The tacrolimus trough concentration in our patient was slightly higher than expected. The infectious route of this pathogen has not been clarified. Imafuku et al. [7] recently speculated that translocation from the intestine to the blood vessels induced secondary bacteremia with several of the symptoms described in these cases, as *H. cinaedi* bacteremia developed after colonic perforation in two of four cases.

In general, the distinct clinical features of *H. cinaedi* bacteremia include fever, enterocolitis, diarrhea, cellulitis, arthritis, meningitis, and recurrent bacteremia. Cellulitis in this condition has the distinct characteristic features of multifocal salmon-pink skin discoloration with no wound infection [6]. In our case, such skin discoloration with swelling and spontaneous pain was evident. *H. cinaedi* infection should thus be included in the differential diagnosis of multifocal cellulitis accompanied by fever in kidney transplant recipients.

The detection of *H. cinaedi* is difficult for two reasons: (1) 4–10 (median, 5) days are required for the detection of these slow-growing pathogens in blood culture, and (2) they grow in film-like shapes and are likely to go unnoticed [6]. The reported lengths of time required for detection of *H. cinaedi* in kidney transplant recipients are 5, 7, 4, 3, and 4 days; in our case, 4 days were required. Therefore, Araoka et al. [8] speculated that the observation period should be extended to at least 7 days when a clinician suspects infection with this pathogen. In addition, Kawamura et al. [6] pointed out differences in detection sensitivity among blood culture systems; the VersaTREK system^®^ (Kohjin Bio, Saitama, Japan) is superior to the Bactec system, which we used. The former system can detect the pathogen within 3 days, whereas the latter system requires 4–10 days.

We performed additional assays to identify the infecting strain in our case because the commercially available detection kit has limitations: few strains are included in the kit, and the identification rate of the kit requires improvement. The accuracy of 16S rRNA gene sequence analysis is also limited because of the high degree of similarity among closely related *Helicobacter* species [9]. GyrB-targeted PCR is more specific for *H. cinaedi* [8]. MALDI-TOF MS has proven over the years to be a simple, rapid, and accurate universal method for the identification of microorganisms, including *H. cinaedi* strains [9].

Minimal preparation is required for MALDI-TOF MS [10]. Samples are overlaid with a suitable matrix (alpha-cyano-4-hydroxycinnamic acid in 50% acetonitrile and 2.5% trifluoroacetic acid) and left for a few minutes to dry before being subjected to the mass spectrometry. After adding the organic matrix to the biomolecules, co-crystallization proceeds rapidly in a spatial array on the target plate. The size and intensities of the peaks of the detected molecules depend on the matrix selected. Then the mixed sample receives a fixed, pulsed laser beam. The part of the matrix gets heated and the mixed sample become excited and ionized states. Ionized molecules are accelerated electronically and converted from TOF measurements to mass/charge values. The result is shown as a spectrum. Each spectrum corresponds to a molecular fragment of the cell during laser desorption. The same bacterium can yield different mass spectra. Protein mass patterns can be used for identification of bacteria at the genus and species levels [11]. Brucker Corporation has an algorithm for phylogenetic analysis, which has not been published. Many studies have shown that MALDI-TOF MS decreases both the time to bacterial identification and the time to effective antibiotic therapy [12, 13]. Regarding the rapidity of identification of bacteria, Seng et al. [14] estimated a mean time to identification by MALDI-TOF MS of 6 min, whereas conventional techniques require 3–48 h. The cost of bacterial identification by MALDI-TOF MS was estimated by Seng et al. [14] to be 17–32% of that of conventional identification methods.

In our case, MALDI-TOF MS showed positivity for *H. cinaedi* earlier than *gyrB*-targeted PCR, enabling us to determine rapidly that a 6-week period of antibiotic therapy was appropriate.

Although no report has described the isolation of the same *H. cinaedi* strain from a human patient and his/her pet, *H. cinaedi* infection is thought to be zoonotic [6]. In our case, subsequent *gyrB*-targeted PCR analysis of a stool sample from the patient’s pet yielded negative results. Taniguchi et al. [9] speculated that *H. cinaedi* comprises two different lineages defined by HSP60 sequences: (1) cluster A from animals and (2) cluster B from humans. We examined the HSP60 sequence of the patient’s blood culture and identified cluster B *H. cinaedi*. Taken together, these findings suggest that our patient was not infected from his dog.

The reason that a blood culture obtained at 11 days was positive and a culture obtained at 15 days was negative in our case is unclear. We speculate that complete eradication of *H. cinaedi* required a prolonged period of antibiotic treatment, possibly because of infection of an atherosclerotic plaque. Khan et al. [15] reported detection of *H. cinaedi* antigen in atherosclerotic plaques in postmortem human specimens.

No guideline for the antibiotic treatment of *H. cinaedi* infection has been established. Because many reports indicate that *H. cinaedi* infection is likely to recur, a 2- to 6-week duration of antibiotic therapy has been recommended. Although one report described positive results of selective decontamination of the digestive tract [7], even in cases of initial *H. cinaedi* infection, the effectiveness of this eradication therapy remains undetermined.

## Conclusions

We have described a rare case of *H. cinaedi* bacteremia with cellulitis in a living-donor kidney transplant recipient, which was identified by MALDI-TOF MS. In this paper, we wish to emphasize the importance of rapid and accurate identification of the pathogenic bacterium. A positive blood culture of gram-negative spiral bacilli from immunocompromised patients is suggestive of infection by *H. cinaedi*, and in such cases the bacterium should be identified rapidly. *H. cinaedi* infection should be included in the differential diagnosis of multifocal cellulitis with fever, especially in immunocompromised patients. MALDI-TOF MS is a simple, rapid, and accurate method of *H. cinaedi* detection.

## Abbreviations

gyrB: gyrase subunit B
PCR: polymerase chain reaction
MALDI-TOF MS: matrix-assisted laser desorption ionization–time-of-flight mass spectrometry
HSP60: heat shock protein 60
